# From mechanical adaptation to innate immune reprogramming in osteoarthritis: a load-immunity framework

**DOI:** 10.3389/fimmu.2026.1931112

**Published:** 2026-08-13

**Authors:** Shiguo Zuo, Lijun He, Zhiying Hou, Quanliang Tian, Yilong Yang, Weichen Huang

**Affiliations:** 1 Department of Spine Surgery, Qianxinan Autonomous Prefecture Hospital of Traditional Chinese Medicine, Xingyi, Guizhou, China; 2 Department of Ultrasound, The Second Affiliated Hospital of Army Medical University, Chongqing, China; 3 Department of Orthopedics, Qianxinan Autonomous Prefecture Hospital of Traditional Chinese Medicine, Xingyi, Guizhou, China; 4 Department of Orthopedics, The Second Affiliated Hospital of Guizhou University of Traditional Chinese Medicine, Guiyang, Guizhou, China

**Keywords:** cGAS-STING, innate immune reprogramming, mechanical loading, mechanoinflammation, osteoarthritis, Piezo1, sterile inflammation

## Abstract

Osteoarthritis (OA) is a whole-joint disease in which mechanical exposure and low-grade sterile inflammation interact, but their coexistence does not by itself explain why a normally adaptive response becomes self-sustaining. This Mini Review therefore focuses on a narrower question: how do specific loading conditions push joint-resident cells from reversible mechanoadaptation into persistent innate immune dysregulation? We define innate immune reprogramming operationally as a load-induced change in the activation threshold, metabolic state, secretory output, or intercellular behavior of a resident or recruited cell that outlasts the initiating mechanical episode or changes its response to subsequent loading. We organize the evidence into three levels: initiating mechanical events; intracellular amplification through calcium overload, mitochondrial stress, NLRP3, and cyclic GMP-AMP synthase (cGAS)-stimulator of interferon genes (STING) signaling; and tissue-specific consequences propagated through interactions among chondrocytes, macrophages, fibroblast-like synoviocytes (FLS), subchondral bone cells, and sensory neurons. Piezo1, TRPV4, NLRP3, and cGAS-STING are treated as context-dependent nodes rather than uniformly pathogenic switches. We also distinguish causal preclinical experiments from human association studies and from proposed joint-level networks. Recent evidence that mitochondria-derived extracellular vesicles released by infrapatellar fat pad mesenchymal stromal cells can transfer mitochondrial DNA (mtDNA) to chondrocytes illustrates how mitochondrial danger signals may propagate extracellularly, although its quantitative importance in human OA remains unresolved. Translationally, load correction has the strongest immediate rationale, whereas molecular interventions require tissue-specific delivery, disease-stage selection, pharmacodynamic biomarkers, and preservation of physiological mechanoadaptation.

## 1 Introduction: the transition from adaptation to persistent dysregulation

Osteoarthritis (OA) is a heterogeneous disease of the entire synovial joint rather than a disorder of cartilage wear alone. Cartilage, synovium, infrapatellar fat pad, subchondral bone, ligaments, muscles, blood vessels, and nerves contribute differently across individuals and disease stages (1, 2). Synovitis and effusion are associated with progression and symptoms in some phenotypes, while macrophage- and fibroblast-derived mediators influence tissue remodeling and pain (3). Mechanical loading is equally fundamental, yet physiological intermittent loading supports matrix renewal, muscle function, and joint adaptation. Pathology emerges when load magnitude, shear, distribution, or recovery time exceeds the adaptive capacity of a particular tissue (4). Aging-associated inflammation and obesity-related metabolic stress can narrow that capacity and make the same external load biologically different across patients (5, 6). Cellular senescence can further reduce adaptive reserve (7).

Previous reviews established that mechanical injury can directly activate inflammatory signaling in cartilage and summarized cartilage mechanosignaling and mechanoresponsive therapeutic concepts (8, 9). A more recent review cataloged parallel mechanical and inflammatory pathways (10). The present review does not use “mechanoinflammation” as shorthand for the simple coexistence of mechanical stress and inflammation. Instead, the load-immunity framework asks when and where a mechanical input changes the future behavior of joint cells: Does the response resolve when the load is removed, or does it persist through altered channel sensitivity, mitochondrial danger signaling, secretory programs, or multicellular feedback? This emphasis on persistence, state dependence, and altered responses to subsequent loading is the intended conceptual advance beyond a pathway inventory.

Here, “innate immune reprogramming” is used only when a mechanical cue changes a cell’s response threshold or functional state beyond the immediate stimulus. Candidate manifestations include inflammation-dependent sensitization of Piezo1 and sustained mitochondrial or senescence-associated stress programs (11, 12). Altered macrophage efferocytosis or metabolic state and fibroblast-like synoviocyte (FLS) programs that continue to recruit or activate myeloid cells are additional candidates (13, 14). Acute NF-κB phosphorylation or cytokine release alone is pathway activation, not proof of reprogramming. Reversibility is therefore central: some changes normalize after unloading or resolution, whereas senescence, matrix remodeling, epigenetic change, or continued cell-cell feedback may maintain the response. Human cross-sectional single-cell data identify states but cannot establish their mechanical origin, duration, or reversibility (15, 16).

These candidate manifestations differ in how readily they reverse once the mechanical environment normalizes. Acute Ca²^+^ influx through Piezo1 and immediate signaling through NF-κB and mitogen-activated protein kinase (MAPK) are generally expected to be more reversible than senescence-, epigenetic-, or matrix-remodeling-associated states (7, 11). However, their resolution kinetics after unloading remain insufficiently defined *in vivo*. IL-1α-dependent sensitization of Piezo1 appears to track the local inflammatory state and may therefore be conditionally reversible (11). However, normalization after resolution of that state has not been directly tested by unloading experiments *in vivo*. Cellular senescence and its associated secretory phenotype are among the least reversible candidates, since they can persist after the initiating mechanical episode and continue to shape the tissue’s response to subsequent loading independent of the original stimulus (7). Placing candidate mechanisms along this reversibility spectrum, rather than treating “reprogramming” as a single binary state, is necessary to distinguish which changes represent a genuine departure from physiological mechanoadaptation.

**Figure 1:**
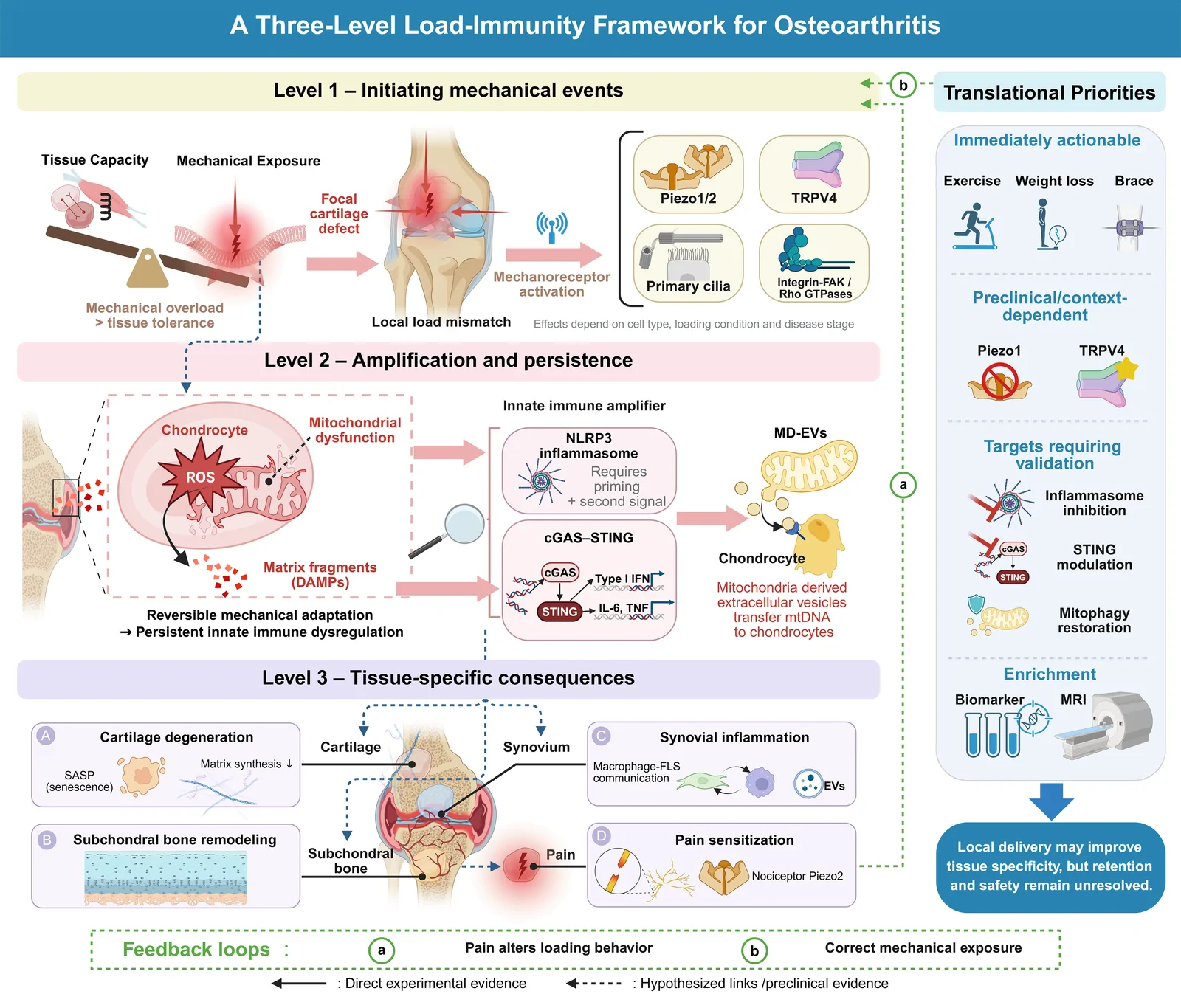
A three-level load-immunity framework for osteoarthritis (physiological loading supports reversible mechanoadaptation. Pathological progression becomes more likely when load exceeds tissue capacity and calcium/mitochondrial stress engages innate immune amplifiers. Persistent cellular states and inter-tissue feedback then reshape the response to subsequent loading. The framework separates context-dependent sensors from downstream amplification and whole-joint consequences; it does not imply that every pathway is active in every patient or disease stage).

The review therefore examines two related issues. The first is the distinction between events that plausibly initiate the transition from physiological mechanoadaptation to pathological innate immune signaling and pathways that mainly amplify an already injured state. The second is the distinction between directly demonstrated tissue-to-tissue interactions and proposed joint-level networks. **Figure 1** and the following sections organize these issues according to a three-level hierarchy comprising loading and sensing, the stress-to-immunity transition, and joint-level persistence.

## 2 A load-immunity framework and an evidence hierarchy

The framework begins with a mismatch between mechanical exposure and tissue capacity (4, 9). Exposure includes compression, tension, hydrostatic pressure, fluid shear, and osmotic change; capacity is set by matrix integrity, cell age, metabolic state, prior inflammation, and recovery time (4, 6). A focal cartilage defect can concentrate stress at its edge while unloading its center, and malalignment can overload one compartment while underloading another (4, 17). Consequently, a single joint contains spatially discordant mechanical environments. Molecular measurements without matched biomechanical localization can therefore obscure rather than clarify load-responsive biology.

The second level is a stress-to-immunity transition. Excessive mechanosensing can engage innate immune pathways through Piezo1-NLRP3 signaling and through mitochondrial dysfunction followed by release of mitochondrial DNA (mtDNA), a damage-associated molecular pattern (DAMP) that activates cGAS-STING (18, 19). This transition is not a single checkpoint: NF-κB and MAPK signaling can occur rapidly after injury, whereas NLRP3 and cGAS-STING may require priming, organelle damage, or sustained nucleic-acid exposure (18, 20). A pathway may therefore initiate a response in one context and amplify it in another.

The third level is persistence across cells and tissues. A transient response can support debris clearance and repair, but a response becomes pathologically reprogrammed when altered cellular states, failed resolution, matrix damage, or reciprocal signaling changes the response to the next mechanical episode (5, 7). For critical interpretation, we classify evidence as: (i) causal preclinical evidence, in which load or a molecular node is experimentally manipulated in cells, explants, or animals; (ii) human associative evidence from tissue, fluid, imaging, or single-cell studies; and (iii) proposed network links inferred by combining datasets or adjacent models. This hierarchy prevents a plausible whole-joint diagram from being mistaken for a fully demonstrated causal chain.

## 3 Initiating mechanical events: context-dependent sensing rather than uniformly pathogenic channels

Chondrocytes receive mechanical inputs after they have been filtered by the extracellular and pericellular matrices. Piezo1 and Piezo2 confer high-strain mechanosensitivity (21, 22). Transient receptor potential vanilloid 4 (TRPV4) responds strongly to dynamic compression and osmotic cues (23, 24). These channels should not be assigned fixed “harmful” or “protective” labels. Piezo1-mediated calcium entry becomes damaging under excessive strain, prolonged activation, matrix loss, or inflammatory sensitization (25, 26). IL-1α can increase Piezo1 expression and lower the threshold for a subsequent load (11). This feed-forward sensitization is a strong candidate for reprogramming, but most causal evidence derives from chondrocyte and experimental OA systems rather than prospective human studies (11, 25).

TRPV4 illustrates the opposite interpretive problem. Under defined dynamic or osmotic loading, TRPV4-dependent calcium signals support anabolic matrix responses (23). Within chondrocytes, its effects nevertheless vary with stimulus pattern and inflammatory background, and transcriptomic analyses show overlapping but distinct Piezo1- and TRPV4-responsive programs (24, 27). Thus, Piezo1 blockade or TRPV4 activation cannot be generalized across cartilage, synovium, bone, and sensory tissues without specifying the loading protocol and disease stage (21, 27).

Primary cilia, integrin-focal adhesion kinase (FAK) complexes, Rho-family GTPases, and actomyosin tension provide additional routes by which cells sense load and matrix stiffness (9, 28). Cilia abundance alone is not a readout of dysfunction: YAP activation can reduce cilia expression while suppressing IL-1β-driven cartilage breakdown (28, 29). Load-activated β1 integrins and fibroblast growth factor receptors regulate partly distinct chondrocyte programs (30). Pericellular matrix degradation changes force transmission to all of these sensors (31). Related mechanosensing machinery has also been described in synovial and subchondral bone compartments, but activation thresholds and outputs are tissue-specific (17, 18). The initiating event is therefore best defined as a context-specific failure of mechanoadaptation, not activation of one universal receptor.

## 4 Amplification and persistence: mitochondrial danger, NLRP3, and cGAS-STING

When loading exceeds tissue capacity, sustained calcium entry and cytoskeletal stress can converge on mitochondrial dysfunction (12, 19). Experimental overload links Piezo1 to NF-κB/NLRP3 signaling, synovitis, and fibrosis, and links Piezo1-dependent mitochondrial injury to mtDNA release and cGAS-STING activation (18, 19). Mitochondrial protection can reduce overload-induced chondrocyte senescence, supporting an upstream role for organelle stress rather than a purely late consequence of tissue destruction (12). However, the strength of the chain differs by step: mechanical perturbation and downstream signaling are causal in preclinical models, whereas the dominant initiating route and its prevalence in human OA remain uncertain (18, 19).

### 4.1 NLRP3 is an amplifier with cell- and stage-specific effects

NLRP3 activation generally requires inflammatory priming plus a second signal such as ionic flux, mitochondrial reactive oxygen species, or organelle damage (32, 33). Restoring AMP-activated protein kinase (AMPK) activity or disrupting the IRF1/GBP5 program reduces inflammasome-associated chondrocyte injury in experimental systems (32, 33). Neuronal NLRP3 can maintain inflammatory and OA pain in mice (34). These findings support causal roles in selected compartments, not a universal master switch. NLRP3-associated cytokines can participate in host defense, debris handling, and repair; the relevant outcome depends on timing, cell type, and persistence (5). This context dependence cautions against assuming that a downstream inflammasome readout identifies a dominant, drug-responsive driver in unselected OA.

### 4.2 cGAS-STING links intracellular injury to extracellular propagation

Cytosolic double-stranded DNA, including leaked mtDNA, is detected by cyclic GMP-AMP synthase (cGAS), which signals through STING, TBK1, IRF3, type I interferon programs, and NF-κB-related outputs (20). TRPM2-dependent calcium signaling may reinforce a calcium-cGAS-STING-NF-κB loop in chondrocytes (35). As with NLRP3, cGAS-STING activity is not intrinsically synonymous with pathology: transient nucleic-acid sensing may contribute to stress surveillance, whereas prolonged activation in a poorly resolving compartment can amplify catabolism or senescence (20, 35). Current OA evidence is dominated by cell and animal models, and pathway activity, cellular source, and disease-stage dependence require validation in human tissue (20, 35).

Mitochondrial danger can also propagate between cells. A recent study found that OA synovial fluid was enriched in TOMM20-positive mitochondria-derived extracellular vesicles containing mtDNA and traced a candidate source to infrapatellar fat pad (IPFP) mesenchymal stromal cells; the study termed these vesicles mitochondria-derived vesicles (MDVs) (36). These extracellular vesicles transferred mtDNA to chondrocytes, activated cGAS-STING-related signaling, suppressed matrix synthesis, and worsened cartilage damage and synovitis after intra-articular administration in rats; STING inhibition attenuated these effects (36). The combination of human fluid association, cell-transfer experiments, and animal perturbation makes extracellular mtDNA transfer a plausible amplification route. It does not yet establish that this route initiates common human OA or quantify its contribution relative to intracellular mtDNA leakage, free extracellular DNA, or other DAMPs.

Together, NLRP3 and cGAS-STING are better positioned as conditional amplifiers (18, 20). They may convert a spatially limited mechanical injury into a longer-lived secretory and intercellular response (19, 36). Whether that response resolves depends on mitochondrial quality control, clearance of dying cells and DNA, matrix repair, and removal or redistribution of the abnormal load (12, 14).

## 5 Tissue-specific consequences: demonstrated interactions versus proposed networks

In cartilage, mechanical injury can reduce matrix synthesis and increase matrix metalloproteinases (MMPs), ADAMTS proteases, chemokines, alarmins, and lipid mediators (4, 37). Senescent chondrocytes can maintain a senescence-associated secretory phenotype (SASP) after the initiating load has ceased (7). Cartilage-synovium co-culture studies directly demonstrate bidirectional effects, and paired transcriptomic data support communication between these tissues (37). These experiments establish that soluble or vesicular signals can cross compartments; they do not establish the direction, timing, or dominance of the same interaction in an intact human joint.

Human single-cell studies demonstrate heterogeneity among synovial macrophages, fibroblasts, endothelial cells, and lymphocytes, with substantial donor-to-donor variation (15). Human atlas data implicate APOE-related communication between synovium and infrapatellar fat pad (16). Mouse single-cell and lineage-tracing experiments demonstrate shared mesenchymal progenitors and coordinated tissue remodeling (38). Together, the human single-cell datasets support the view that the M1/M2 macrophage dichotomy is inadequate, but most available atlases are cross-sectional and do not prove that mechanical loading produced a particular state (15, 16). Longitudinal sampling or controlled mechanical challenge is needed to test whether a state is transient, reversible, or durably reprogrammed.

Several macrophage-FLS interactions are experimentally supported. OA FLS occupy inflammatory and matrix-remodeling states and can secrete R-spondin 2 (39). FLS-derived extracellular vesicles can promote macrophage glycolysis and inflammatory activation (13). YAP1-dependent FLS glycolysis can enhance macrophage recruitment in metabolically stressed experimental OA (40). Macrophage polarization can increase R-spondin 2-mediated damage in models (41). Reduced GAS6 impairs macrophage efferocytosis and worsens obesity-associated OA (14). Post-traumatic single-cell data also show rapid expansion and state change of monocyte-macrophage populations (42). By contrast, a closed joint-wide loop in which a defined mechanical cue sequentially reprograms FLS, macrophages, cartilage, bone, and nerves has not been demonstrated in humans. That network should remain a testable model rather than a settled mechanism.

Subchondral bone provides additional tissue-specific consequences. Mechanical disturbance can alter osteoclast-osteoblast coupling, microdamage repair, vascular invasion, and osteochondral signaling (17, 43). Osteoclast-precursor-derived platelet-derived growth factor-BB promotes pathological angiogenesis in experimental OA (44). Preserving a physiological hypoxic niche can limit subchondral deterioration (45). PGE2-EP4 signaling links osteoclast activity to joint pathology (46). These pathways are mechanistically credible but are not yet integrated into a single temporally resolved load-immunity sequence. Mast-cell and neutrophil contributions may be important in selected phenotypes, but their causal roles in common OA are less established (3, 5).

OA therefore differs from rheumatoid arthritis not because immune activity is absent, but because it usually lacks sustained autoantigen-driven adaptive synovitis and instead shows heterogeneous, often intermittent, low-grade innate and stromal responses associated with damage sensing, debris clearance, repair, fibrosis, and metabolic stress (3, 5). The same macrophage or fibroblast state may be protective after microinjury yet damaging when prolonged or misplaced (5, 14). Functional labels such as efferocytosis, matrix production, lipid handling, angiogenic support, or nociceptor communication are consequently more informative than a simple pro-inflammatory/anti-inflammatory binary.

## 6 Pain as a tissue-specific consequence, not a surrogate for cartilage loss

Synovitis, bone marrow lesions, osteochondral remodeling, fibrosis, and neurovascular ingrowth can all contribute to pain (47). Because articular cartilage is a neural, nociceptive input more often arises from synovium, subchondral bone, periosteum, ligaments, fat pads, and muscle (47). This distribution helps explain why radiographic structural severity and patient-reported pain correlate imperfectly (47).

Sensory neurons also participate in mechanosensing. Nociceptor-specific Piezo2 deletion reduces mechanical sensitization in experimental OA, and some Piezo2-positive neurons express the nerve growth factor (NGF) receptor TrkA (48). NGF, PGE2, cytokines, tissue acidosis, and neurovascular ingrowth can lower peripheral thresholds, while sustained input promotes central sensitization (47, 49). The efficacy of tanezumab supports the NGF pain axis, while the occurrence of rapidly progressive joint damage in some treated patients highlights a potential load-immunity trade-off; one interpretive hypothesis is that strong analgesia may reduce protective pain behavior without correcting the mechanical environment, although this mechanism was not directly tested (49). Pain mechanisms should therefore be phenotyped alongside tissue activity rather than treated as a direct measure of inflammatory or structural progression.

## 7 Translational priorities: plausible targets, narrow windows, and biomarker constraints

The most immediately actionable strategy is to correct modifiable mechanical exposure while preserving beneficial loading, as summarized in **Table 1** (50, 51). Exercise, strength training, weight loss, gait retraining, bracing, and management of instability can reduce excessive or poorly distributed stress (50). In the IDEA trial, diet plus exercise reduced knee load and inflammatory markers while improving symptoms in overweight and obese adults with knee OA (51). These interventions are clinically feasible, but the therapeutic dose is individualized: prolonged unloading can impair muscle and cartilage adaptation, whereas inadequately calibrated exercise can exceed the capacity of an actively inflamed or unstable joint (50).

**Table 1:** Mechanoinflammatory nodes ranked by role, evidence, and translational constraint.

Node/transition	Main context	Role in hierarchy	Evidence and uncertainty	Translational implication
Mechanical exposure	Whole joint	Initiator when exposure exceeds tissue capacity	Clinical and biomechanical evidence is strong; dose and spatial distribution vary by patient (4, 50).	Correct malalignment and instability, reduce excessive loading, and allow adequate recovery while preserving beneficial activity
Piezo1/TRPV4	Cartilage (best-established evidence)	Context-dependent sensing; Piezo1 can amplify high strain, whereas TRPV4 can support adaptation	Causal preclinical data; limited human target-engagement evidence; effects depend on load, cell, and stage (21, 27).	Prefer local, transient, or load-contingent modulation; avoid nonselective systemic channel inhibition
Mitochondrial stress/mtDNA	Chondrocytes; IPFP; synovial compartment	Bridges mechanical stress to innate immune amplification; may propagate through extracellular vesicles	Cell and animal causality plus human fluid association; quantitative importance in human OA unknown (12, 36).	Protect mitochondrial quality control and define intracellular versus extracellular routes
NLRP3	Chondrocytes, macrophages, sensory neurons	Conditional amplifier after priming and danger signals	Causal in selected models; heterogeneous across compartments and stages; downstream IL-1 targeting has shown limited and inconsistent clinical efficacy (34, 52).	Require pathway-active enrichment, timing, and preservation of repair and host defense
cGAS-STING	Chondrocytes and other stressed joint cells	DNA-sensing amplifier of persistent stress programs	Predominantly preclinical; human activity and stage dependence not established (20, 35).	Use local delivery and pharmacodynamic markers; avoid assuming uniform pathogenicity
Macrophage-FLS communication	Synovium and adjacent fat pad	Intercellular maintenance and spread	Human heterogeneity and several pairwise interactions are demonstrated; closed joint-wide loops remain inferred (13, 15).	Target functions or states rather than stable M1/M2 labels
Pain/neuroimmune signaling	Synovium, osteochondral unit, sensory nerves	Tissue-specific outcome and behavioral feedback on loading	Human analgesic efficacy supports NGF axis; structural safety signals expose trade-offs (49).	Relieve pain while monitoring load behavior and structural risk

Direct mechanosensor targeting remains preclinical and context-sensitive (21, 27). Piezo1 inhibition might reduce high-strain calcium overload, but systemic blockade could affect vascular, sensory, and other mechanically responsive tissues (21, 25). In chondrocytes, TRPV4 activation may support cartilage anabolism under defined loading, whereas its downstream effects vary with loading conditions and cellular context (23, 27). YAP/TAZ, fibroblast growth factor receptors, and β1 integrins likewise regulate context-dependent chondrocyte responses (29, 30). Local delivery, transient exposure, or downstream modulation may widen the therapeutic window, but each approach still requires evidence that physiological mechanoadaptation is preserved (9, 52).

The same critical standard applies to NLRP3, cGAS-STING, mitochondrial, and macrophage-FLS strategies. Mitochondrial antioxidants, restoration of mitophagy, inflammasome inhibition, STING modulation, senomorphic agents that suppress deleterious SASP programs, and interruption of immune-stromal amplification all have a mechanistic rationale (20, 52). Their major barriers are incomplete human target validation, uncertain timing, redundant inflammatory pathways, cell-state plasticity, and the possibility of impairing host defense, debris clearance, or repair (5, 52). The limited and inconsistent efficacy of IL-1 blockade in unselected OA provides a general warning that a biologically active pathway may not be dominant, stage-appropriate, or safely druggable (52). Intra-articular hydrogels, nanoparticles, and extracellular-vesicle platforms may improve local exposure, but retention, manufacturing consistency, off-target uptake, and repeated-dosing safety remain unresolved (52, 53).

No single validated biomarker currently identifies an active load-immunity circuit (52, 54). Aging-related synovial gene-expression signatures have been proposed as candidate biomarkers (54). However, their ability to identify such a circuit has not been validated. A plausible enrichment strategy would combine mechanical exposure (lower-limb alignment plus gait kinetics and kinematics); anatomical activity (ultrasound or contrast-enhanced magnetic resonance imaging (MRI) synovitis, effusion, and bone marrow lesions); and pathway-proximal measures (selected DAMPs, mtDNA, inflammatory lipids, or cell-state signatures in synovial fluid or tissue). Serum measurements are more scalable but dilute compartment-specific signals; synovial fluid is more local but cannot be obtained repeatedly from every joint. Standardized activity challenges with short-interval sampling are conceptually attractive for testing reversibility and pharmacodynamic response, but remain research tools rather than established clinical biomarkers.

## 8 Challenges and decisive experiments

The central measurement problem is temporal alignment. Joint load varies from step to step, while tissue sampling usually provides a single late snapshot (4, 15). Wearable sensors, instrumented gait analysis, quantitative MRI, and finite-element modeling can estimate patient-specific exposure only if their measurements are linked in time and space to molecular readouts. Eroded and intact cartilage, inflamed and quiescent synovium, and bone marrow lesions can coexist within the same knee; pooled sampling can erase the local relationship between load and cell state (1, 3).

Single-cell and spatial studies should therefore be integrated with mapped biomechanics rather than interpreted as stand-alone atlases (15, 16). Future studies should report loading waveform, magnitude, frequency, duration, and recovery; cell and tissue compartment; sex, age, and metabolic state; disease stage; and the timing of sampling. Animal models remain essential for causal perturbation but compress years of human OA into weeks and often overrepresent post-traumatic disease (4, 42). Replication in human explants, longitudinal fluid-sampling or imaging studies, and intervention-linked pharmacodynamic samples is needed before a proposed pathway is considered a human disease driver.

Three experiments would most directly test the framework. First, determine whether removing or normalizing load reverses Piezo1 sensitization, mitochondrial stress, and defined cell states, and over what time scale (11, 12). Second, use lineage-resolved perturbation to establish whether intracellular mtDNA leakage and extracellular-vesicle-mediated mtDNA transfer are parallel, redundant, or stage-specific routes to cGAS-STING activation (19, 36). Third, prospectively test whether a combined mechanical-anatomical-molecular signature predicts response to a pathway-specific intervention better than conventional OA severity (52, 54). These studies would distinguish transient activation from reprogramming and convert a proposed joint network into a falsifiable causal model.

## 9 Conclusion

Mechanoinflammation does not redefine all of OA, and it is more than the simultaneous presence of abnormal load and inflammation. Its most useful interpretation is the transition from reversible mechanoadaptation to a persistent change in how joint cells sense, amplify, and remember mechanical injury. The best-supported initiating events are context-dependent mechanical and calcium stresses (11, 25). Mitochondrial dysfunction, NLRP3, cGAS-STING, and mtDNA-containing extracellular vesicles are candidate amplifiers whose importance varies by cell type, compartment, loading condition, and disease stage (18, 36). The central unresolved question is which cellular transition first becomes non-resolving in human OA and whether correcting the mechanical environment can still reverse it. Answering that question will require temporally matched biomechanics and tissue biology, rather than broader pathway lists, and therapies that suppress pathological persistence without abolishing physiological repair.
